# Expanding the phenotypic spectrum of *BCS1L*‐related mitochondrial disease

**DOI:** 10.1002/acn3.51470

**Published:** 2021-10-18

**Authors:** Omar Hikmat, Pirjo Isohanni, Nandaki Keshavan, Matteo P. Ferla, Elisa Fassone, Mary‐Alice Abbott, Marcello Bellusci, Niklas Darin, David Dimmock, Daniele Ghezzi, Henry Houlden, Federica Invernizzi, Nazreen B. Kamarus Jaman, Manju A. Kurian, Eva Morava, Karin Naess, Juan Darío Ortigoza‐Escobar, Sumit Parikh, Alessandra Pennisi, Giulia Barcia, Karin B. Tylleskär, Damien Brackman, Saskia B. Wortmann, Jenny C. Taylor, Laurence A. Bindoff, Vineta Fellman, Shamima Rahman

**Affiliations:** ^1^ Department of Paediatrics and Adolescent Medicine Haukeland University Hospital Bergen 5021 Norway; ^2^ Department of Clinical Medicine (K1) University of Bergen Norway; ^3^ Children's Hospital University of Helsinki and Helsinki University Hospital Helsinki Finland; ^4^ Stem Cells and Metabolism Research Program Faculty of Medicine University of Helsinki Helsinki Finland; ^5^ Mitochondrial Research Group UCL Great Ormond Street Institute of Child Health London UK; ^6^ Metabolic Unit Great Ormond Street Hospital for Children NHS Foundation Trust London UK; ^7^ NIHR Oxford Biomedical Research Centre Wellcome Centre for Human Genetics University of Oxford Oxford UK; ^8^ Medical Genetics Department of Pediatrics UMass Chan Medical School Baystate USA; ^9^ Reference Center for Hereditary Metabolic Disorders ‐ MetabERN ‘12 de Octubre’ University Hospital Madrid Spain; ^10^ Instituto de Investigación Hospital 12 de Octubre (imas12) Madrid Spain; ^11^ Department of Pediatrics University of Gothenburg The Queen Silvia Children's Hospital Gothenburg Sweden; ^12^ Rady Children’s Institute for Genomic Medicine San Diego California USA; ^13^ Unit of Medical Genetics and Neurogenetics Fondazione IRCCS Istituto Neurologico Carlo Besta Milan 20126 Italy; ^14^ Department of Pathophysiology and Transplantation University of Milan Milan Italy; ^15^ Department of Molecular Neuroscience UCL Queen Square Institute of Neurology London United Kingdom; ^16^ Neurogenetics Group Developmental Neurosciences Zayed Centre for Research into Rare Diseases in Children UCL Great Ormond Street Institute of Child Health London UK; ^17^ Department of Clinical Genomics Mayo Clinic Rochester Minnesota USA; ^18^ Metabolic Center University Hospitals Leuven Leuven 3000 Belgium; ^19^ Centre for Inherited Metabolic Diseases Karolinska University Hospital Stockholm Sweden; ^20^ Department of Medical Biochemistry and Biophysics Karolinska Institutet Stockholm Sweden; ^21^ Movement Disorders Unit Institut de Recerca Sant Joan de Déu CIBERER‐ISCIII Barcelona Spain; ^22^ European Reference Network for Rare Neurological Diseases (ERN‐RND) Barcelona Spain; ^23^ Neuroscience Institute Cleveland Clinic Cleveland OH USA; ^24^ Federation of Medical Genetics and Reference Center for Mitochondrial Diseases (CARAMMEL) Necker ‐ Enfants Malades Hospital Paris France; ^25^ University Children’s Hospital Paracelsus Medical University Salzburg Austria; ^26^ Radboud Center for Mitochondrial Medicine (RCMM) Amalia Children’s Hospital, Radboudumc Nijmegen The Netherlands; ^27^ Neuro‐SysMed Center of Excellence for Clinical Research in Neurological Diseases Department of Neurology Haukeland University Hospital Bergen 5021 Norway; ^28^ Folkhälsan Research Center Helsinki Finland; ^29^ Department of Clinical Sciences Lund University Paediatrics Sweden

## Abstract

**Objective:**

To delineate the full phenotypic spectrum of *BCS1L‐*related disease, provide better understanding of the genotype–phenotype correlations and identify reliable prognostic disease markers.

**Methods:**

We performed a retrospective multinational cohort study of previously unpublished patients followed in 15 centres from 10 countries. Patients with confirmed biallelic pathogenic *BCS1L* variants were considered eligible. Clinical, laboratory, neuroimaging and genetic data were analysed. Patients were stratified into different groups based on the age of disease onset, whether homozygous or compound heterozygous for the c.232A>G (p.Ser78Gly) variant, and those with other pathogenic *BCS1L* variants.

**Results:**

Thirty‐three patients were included. We found that growth failure, lactic acidosis, tubulopathy, hepatopathy and early death were more frequent in those with disease onset within the first month of life. In those with onset after 1 month, neurological features including movement disorders and seizures were more frequent. Novel phenotypes, particularly involving movement disorder, were identified in this group. The presence of the c.232A>G (p.Ser78Gly) variant was associated with significantly worse survival and exclusively found in those with disease onset within the first month of life, whilst other pathogenic *BCS1L* variants were more frequent in those with later symptom onset.

**Interpretation:**

The phenotypic spectrum of *BCS1L‐*related disease comprises a continuum of clinical features rather than a set of separate syndromic clinical identities. Age of onset defines *BCS1L‐*related disease clinically and early presentation is associated with poor prognosis. Genotype correlates with phenotype in the presence of the c.232A>G (p.Ser78Gly) variant.

## Introduction

Disorders of mitochondrial oxidative phosphorylation (OXPHOS) represent one of the most common groups of inherited metabolic diseases, with a combined minimum birth prevalence of 1 in 4300 live births.^1^, ^2^ Clinically, affected individuals can present with a spectrum of heterogeneous phenotypes and disease onset at any time during their life span, and often with multi‐organ involvement. However organs with high energy demand, such as the brain, heart and the skeletal muscles, are the most vulnerable.^3^ Despite the advances in diagnostic methods, early clinical recognition of patients with mitochondrial disorders in general is still challenging.

An important subgroup amongst mitochondrial disorders comprises the mitochondrial complex III (CIII) deficiencies.^4^ CIII (also known as cytochrome bc1 complex or ubiquinol‐cytochrome *c* reductase) catalyses the transfer of electrons from reduced coenzyme Q_10_ to cytochrome *c* whilst simultaneously pumping protons from the mitochondrial matrix across the inner mitochondrial membrane to the intermembrane space.^5^, ^6^ CIII is a multi‐heteromeric enzyme complex consisting of 11 different subunits, of which 10 subunits (core proteins I and II, 6 small subunits, cytochrome c1 and the Rieske FeS protein) are encoded by nuclear genes, whilst the cytochrome b subunit is encoded by mtDNA.^6^, ^7^ Pathogenic variations in several nuclear genes affecting CIII structural subunits (*UQCRB, UQCRQ, UQCRC2*, *UQCRFS1* and *CYC1*) or assembly factors (*TTC19, BCS1L, LYRM7/MZM1L, UQCC2, UQCC3* and *HCCS*) have been reported to be associated with mitochondrial CIII deficiency and human disease.^8^, ^9^

The most frequent cause of mitochondrial CIII deficiencies is due to defects in the *BCS1L* gene encoding BCS1, a mitochondrial inner membrane protein that acts as a translocase for insertion of the Rieske FeS subunit into the precomplex of CIII to facilitate assembly of the holoenzyme complex.^8^, ^10^, ^11^ The two major phenotypes which are well known to be associated with disease‐causing variants in *BCS1L* are GRACILE and Björnstad syndromes. Classical GRACILE syndrome (MIM 603358) is an autosomal recessive disease characterised by **G**rowth **R**estriction, **A**minoaciduria as a sign of tubulopathy, **C**holestasis with **I**ron overload in the liver, **L**actic acidosis and **E**arly death.^12^ This was initially identified as a Finnish heritage disease and is mainly caused by a specific homozygous variant, c.232A>G (p.Ser78Gly), in the *BCS1L* gene.^13^, ^14^ The other less frequently reported phenotype is Björnstad syndrome (MIM 262000) which is characterised by brittle hair (*pili torti*) and sensorineural hearing loss.^15^, ^16^ Other phenotypes that have also been reported include tubulopathy, hepatopathy and encephalopathy^17^ and Leigh‐like syndrome.^18^, ^19^

We aimed in this study to improve the clinical recognition of patients with *BCS1L* disease, provide better understanding of the genotype–phenotype correlations and identify reliable prognostic disease markers using data from the largest known multinational cohort of patients with confirmed biallelic pathogenic *BCS1L* variants.

## Patients and Methods

### Study design and population

We conducted a multinational retrospective study of patients from 15 centres in 10 countries: Finland (Children's Hospital, Helsinki University Hospital), United Kingdom (Great Ormond Street Hospital, London), United States (Department of Clinical Genomics, Mayo Clinic, Rochester, Minnesota; Neuroscience Institute, Cleveland Clinic, Cleveland, Ohio; and Department of Pediatrics, University of Massachusetts Medical School—Baystate, Massachusetts), France (Necker‐Enfants Malades Hospital, Paris), Spain (12 de Octubre University Hospital, Madrid and Hospital Sant Joan de Déu, Barcelona), Sweden (Centre for Inherited Metabolic Diseases, Karolinska University Hospital, Stockholm and The Queen Silvia Children's Hospital and Sahlgrenska University Hospital, Gothenburg), Norway (Haukeland University Hospital), Austria (University Children’s hospital, Salzburg), Italy (Fondazione IRCCS Istituto Neurologico Carlo Besta, Milan) and Oman (Sultan Qaboos University Hospital, Oman). Previously unpublished patients with confirmed biallelic pathogenic *BCS1L* variants who had been diagnosed and followed up at the participating centres were considered eligible.

Detailed clinical, biochemical, muscle biopsy, neurophysiological, neuroimaging and genetic data were obtained using a standardised case report form completed by the responsible investigator(s) at each centre and reviewed by the study principal investigator (O.H). Data entry was completed in December 2020.

The date of disease onset was defined as the date of the first symptom(s) requiring medical evaluation. End of follow‐up was defined as the date of the patient’s last follow‐up visit or death. Available clinical and laboratory longitudinal data, both at disease onset and later during the disease course, were collected. Small for gestational age was defined as newborns with birth weight below the 10th percentile for the gestational age.^20^ Early death was defined as death within the first year of life.

Proximal renal tubulopathy included Fanconi syndrome and was defined as having two or more of the following: generalised aminoaciduria, glycosuria, low molecular weight proteinuria, bicarbonate loss resulting in renal tubular acidosis and renal salt wasting.^21^

Liver involvement was defined by the presence of two or more of the following parameters in at least two different time points: elevation of aspartate aminotransferase (ASAT), alanine aminotransferase (ALAT), gamma‐glutamyltransferase (GGT), bilirubin or ammonia; low serum albumin; or pathological histological findings on liver biopsy.

Movement disorders included dystonia, athetosis and tremor.

### Data analysis

Initially, detailed clinical, laboratory, neuroimaging and genetic data for the whole cohort were analysed to study the phenotypic spectrum of the disease regardless of the age of disease onset or genotype. To study the disease spectrum beyond the neonatal period, the patients were classified into those with disease onset within the first month of life and those with later onset. The study cohort was further stratified into those who were homozygous or compound heterozygous for the c.232A>G (p.Ser78Gly) variant and those with other pathogenic *BCS1L* variants.

### Protein modelling

Models were prepared with PyRosetta^22^ using the cryogenic electron microscopy structures of mouse BCS1 (the protein encoded by *BCS1L*) PDB:6UKP and PDB:6UKS.^23^ The structures were energy minimised against their density maps (EMDB‐20808 and EMDB‐20811) and then used as a template to thread the human sequence (92% identity) of BCS1 using RosettaCM.^24^ The ATPγS was converted into an ATP, the 1–49 span was added as parallel helices for illustrative purposes and then further energy minimised. The difference in Gibbs free energy (∆∆G) was calculated by introducing the mutation and energy minimising in each conformation (single‐point energy). The code used is openly available at https://github.com/matteoferla/BCS1_analysis. Interactive page was created using Michelaɴɢʟo.^25^

### Statistical analysis

Detailed descriptive data analysis was performed using SPSS (Statistical Package of Social Sciences), Version 26.0. A two‐sided *p* value less than 0.05 was considered to be statistically significant. For survival analysis, the endpoint was time to death which was defined as the time from the date of disease onset to the date of death. Univariate survival analysis was performed using the log‐rank test (Kaplan–Meier) to compare differences in survival time between categories.

### Ethical statement

Ethical approval for the study was obtained from the Regional Committee for Medical and Health Research Ethics, Western Norway (REK 2017/625). Each participating centre had obtained approval by the local ethical committee. The study was registered as an audit at Great Ormond Street Hospital, London, UK (Registration Number: 2224).

## Results

### Demography

Thirty‐three patients, (17 male, 16 female) with biallelic pathogenic *BCS1L* variants were identified. Ten were diagnosed in Finland, five in the United Kingdom, four in the United States, three each in France and Sweden, two each in Austria, Norway and Spain and one each in Italy and Oman. The majority of patients were of European ancestry (*n* = 23), whilst three were from the Middle East, three Turkish, two Pakistani and two Black American.

### Phenotypic spectrum

The majority (73%, *n* = 24/33) of patients included in this study cohort had disease onset either at birth or within the first month of life. Fourteen patients were born prematurely with gestational age less than 37 completed weeks and 19 (63%, *n* = 19/30) patients were small for gestational age. A symptom‐free period ranging from 3 months to 7 years was observed in nine patients.

Patients included in this cohort presented with a spectrum of clinical phenotypes including lactic acidosis (*n* = 27), failure to thrive (*n* = 27), proximal renal tubulopathy (*n* = 24), hepatopathy (*n* = 24), early death (*n* = 14), sensorineural hearing loss (*n* = 12), iron overload (*n* = 10), movement disorders (*n* = 9), cholestasis (*n* = 7) and seizures (*n* = 6). Other features observed in our cohort include global developmental delay (*n* = 9), attention deficit hyperactivity disorder (*n* = 3), cataract (*n* = 3), learning difficulties (*n* = 2), optic atrophy (*n* = 1) and hypertrophic cardiomyopathy (*n* = 1). A summary of the clinical phenotypes observed in our cohort is provided in Table 1. Thirteen patients fulfilled the diagnostic criteria for classical GRACILE syndrome (cases 2–10, 24, 27, 32 and 33, Table 1) and two for classical Björnstad syndrome (cases 26 and 28, Table 1).

**Table 1:** Phenotypic spectrum of patients with *BCS1L* disease included in the study cohort.

Case no.	Age [at death]	Growth restriction	Tubulo pathy	Cholestasis	Hepatopathy	Iron overload	Failure to thrive	Lactic acidosis	Early death	*Pili torti*	SNHL	Movement disorders	Seizures	Other features
1	11 y	Yes	No	ND	Yes	ND	Yes	Yes	No	ND	Yes	No	Yes	LD, ataxia
2	[5 d]	Yes	Yes	Yes	Yes	Yes	Yes	Yes	Yes	ND	ND	No	No	
3	[3 d]	Yes	Yes	Yes	Yes	Yes	Yes	Yes	Yes	ND	ND	No	No	
4	[4 d]	Yes	Yes	Yes	Yes	Yes	Yes	Yes	Yes	ND	ND	No	No	
5	[21 d]	Yes	Yes	Yes	Yes	Yes	Yes	Yes	Yes	ND	Yes	No	No	
6	[27 d]	Yes	Yes	Yes	Yes	Yes	Yes	Yes	Yes	No	Yes	No	No	
7	[2 d]	Yes	Yes	Yes	Yes	Yes	Yes	Yes	Yes	ND	ND	No	No	PDA
8	[3 m]	Yes	Yes	ND	Yes	Yes	Yes	Yes	Yes	ND	ND	No	No	
9	[3 d]	YES	ND	ND	Yes	Yes	Yes	Yes	Yes	ND	ND	No	No	
10	[1 d]	Yes	ND	ND	Yes	ND	ND	Yes	Yes	ND	ND	No	No	PND
11	ND	No	Yes	ND	No	ND	No	Yes	ND	ND	No	Yes	No	Ataxia, bulbar palsy
12	[3 y]	Yes	Yes	ND	Yes	ND	Yes	Yes	No	ND	No	No	No	GDD
13	[4.5 y]	No	Yes	ND	Yes	ND	Yes	Yes	No	ND	No	Yes	Yes	GDD,VI
14	11.5 y	No	No	No	No	No	No	Yes	No	No	Yes	Yes	Yes	GDD, ADHD
15	9.5 y	No	Yes	ND	Yes	Yes	Yes	Yes	No	No	Yes	Yes	Yes	GDD,ADHD
16	ND	No	Yes	ND	ND	ND	Yes	Yes	No	ND	ND	Yes	No	Maculopathy, OA, NC
17	24 y	No	Yes	ND	No	ND	Yes	Yes	No	Yes	Yes	Yes	No	Migraine, cataract
18	[1 m]	Yes	Yes	ND	Yes	ND	Yes	Yes	Yes	ND	ND	No	Yes	GDD, cataract,
19	4 y	Yes	No	ND	Yes	ND	Yes	Yes	No	No	ND	NO	No	GDD, LD
20	2 m	ND	Yes	ND	Yes	ND	Yes	ND	No	No	ND	No	No	HCM
21	4 m	Yes	Yes	Yes	Yes	ND	Yes	Yes	No	No	Yes	No	No	Cataract
22	13 y	Yes	Yes	No	No	No	Yes	No	No	No	No	No	No	
23	10 y	No	Yes	No	No	No	Yes	No	No	No	No	No	No	
24	[11 m]	Yes	Yes	ND	Yes	ND	Yes	Yes	Yes	No	No	No	No	
25	[14 y]	ND	Yes	No	Yes	ND	Yes	Yes	No	No	No	Yes	Yes	
26	13.5 y	No	No	No	No	ND	No	No	No	Yes	Yes	No	No	
27	[1 m]	Yes	Yes	ND	Yes	ND	Yes	Yes	Yes	ND	ND	No	No	
28	15 y	No	No	No	No	ND	No	ND	No	Yes	Yes	No	No	
29	ND	No	ND	ND	Yes	Yes	Yes	Yes	No	ND	Yes	No	No	
30	10 y	No	No	ND	No	No	No	No	No	ND	Yes	Yes	No	GDD, anxiety, ADHD
31	[2 y]	Yes	Yes	ND	Yes	ND	Yes	Yes	Yes	Yes	Yes	Yes	No	GDD
32	5 y	Yes	Yes	ND	Yes	ND	Yes	ND	ND	ND	ND	No	No	AIH
33	[15 m]	ND	Yes	ND	Yes	ND	Yes	Yes	Yes	No	ND	No	No	GDD

Key: ADHD: attention deficit hyperactivity disorder, AIH: autoimmune hepatitis, d: days, GDD: global developmental delay, HCM: hypertrophic cardiomyopathy, LD: learning difficulties, m: months, NC: nephrocalcinosis, ND: no data available, OA: optic atrophy, SNHL: sensorineural hearing loss, VI: visual impairment, y: years.

If we stratified patients into those presenting within the first month of life (73%, *n* = 24/33) and those presenting later (27%, *n* = 9/33), we found that features consistent with GRACILE syndrome such as growth failure, lactic acidosis, tubulopathy, hepatopathy, iron overload and early death were more frequently observed in those with onset within the first month of life as compared to those with later onset. In contrast, features such as movement disorders (dystonia, athetosis and tremor) and seizures occurred across all ages but were more frequently identified in our study cohort in those with onset after the first month of life (Table 2). Of note, a paroxysmal movement disorder without evidence of magnetic resonance imaging (MRI) brain lesions suggestive of Leigh syndrome was observed in case 14, who initially presented with sensorineural hearing loss (SNHL) at 8 months of age. By 12–16 months he was noted to have an unsteady gait, and a paroxysmal movement disorder emerged by 18–20 months of age. Paroxysmal episodes lasting approximately 5 min and occurring 4–5 times per day were characterised by increasing unsteadiness of gait with reduced balance and increased falls, sometimes with associated stiffening or posturing on one side of the body. These episodes were precipitated mainly by exertion, such as running, and relieved by rest. MRI brain was normal. This patient also had a severe attention deficit hyperactivity disorder (ADHD) and treatment with clonidine and guanfacine both increased the ataxia and falls. His younger brother (patient 15) also has prominent ataxia, SNHL and ADHD, but not the paroxysmal movement disorder.

**Table 2:** Clinical features, genetic findings and survival analysis for patients with disease onset before and after the age of 1 month.

Age of disease onset	Onset <1 month of age	Onset >1 month of age
Phenotype		
Median age at onset (range)	1 day (1 day–29 days)	11 months (3 months–7 years)
Lactic acidosis	21/22 (95%)	5/8 (62%)
Hepatopathy	21/23 (91%)	3/9 (33%)
Failure to thrive/feeding difficulties	21/23 (91%)	6/9 (67%)
Proximal renal tubulopathy	18/21 (86%)	6/9 (67%)
Growth restriction	17/22 (70%)	2/8 (25%)
Sensorineural hearing loss	6/10 (60%)	6/9 (67%)
Pili tori	0/6 (0%)	4/9 (44%)
Leigh‐like phenotype	1/24 (4%)	1/9 (11%)
Seizures	3/21 (12%)	3/9 (33%)
Movement disorders	4/24 (20%)	5/9 (55%)
Genetic findings		
Homozygous c.232A>G (p.Ser78Gly)	9/24 (38%)	0/9 (0%)
Compound heterozygous c.232A>G (p.Ser78Gly)	4/24 (17%)	0/9 (0%)
Other pathogenic BCS1L gene variants	11/24 (45%)	9/9 (100%)
Survival data		
Survival status ‐ alive	7/22 (32%)	7/9 (78%)
Survival status ‐ deceased	15/22 (68%)	2/9 (22%)
Early death	14/22 (63%)	0/9 (0%)
Median time to death (range)	27 days (1 day–4.5 years)	8 years (2 years–14 years)

### Laboratory and neuroimaging findings

Laboratory investigations revealed elevated serum lactate in the majority of patients (*n* = 26/30, 87%). Elevations of alanine aminotransferase (*n* = 13/24, 54%) and aspartate aminotransferase (*n* = 15/21, 71%) levels were also frequently observed, together with hypoglycaemia (*n* = 13/25, 52%) and hypoalbuminaemia (*n* = 10/17, 59%). Major laboratory findings, both at disease onset and later, are summarised in Table S1.

Respiratory chain enzyme activities (Table S2) showed CIII deficiency (CII+CIII or CIII) in muscle biopsy (*n* = 10/12, 83%), in liver biopsy (*n* = 3/4, 75%) and in cultured skin fibroblasts (*n* = 1/3, 33%).

MRI of the brain was available for 10 cases. The majority (*n* = 8/10, 80%) showed abnormalities at the time the first imaging was performed. The most common cerebral MRI finding was T2/FLAIR hyperintensities scattered in the deep white matter, thalamus and dentate nucleus. Detailed description of the brain MRI findings is provided in Table S3.

### Genetic findings

**Figure 1:**
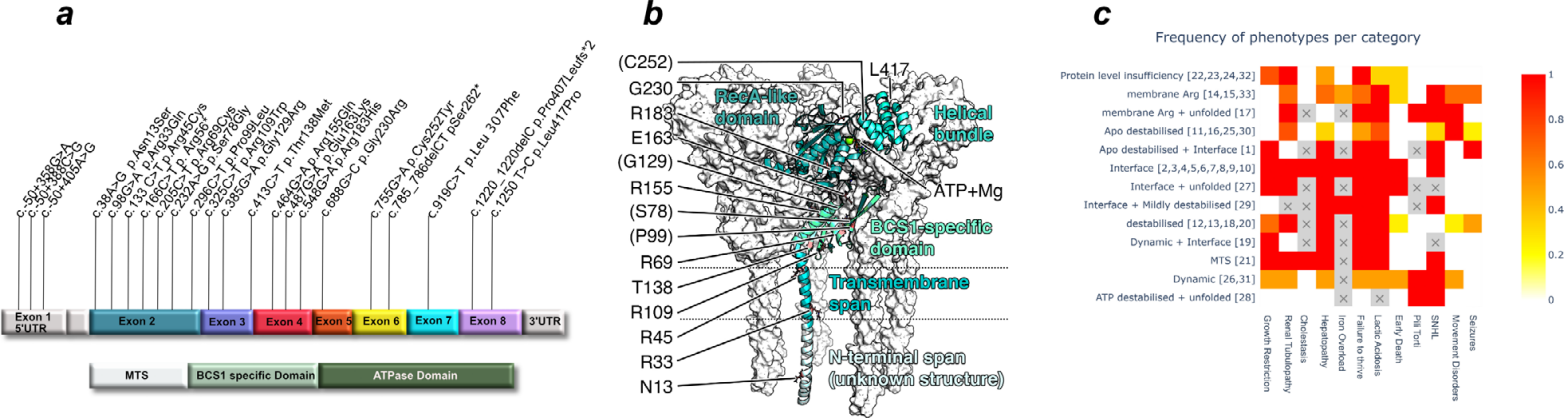
*BCS1L* exon map, secondary structure and phenotype heatmap. (A) Schematic diagram of the *BCS1L* cDNA and protein domains illustrating pathogenic variants identified in this study. aa: amino acid, MTS: mitochondrial targeting sequence and UTR: untranslated region. Pathogenic variants identified were located across all *BCS1L* exons, therefore involving amino acid positions in both functional domains. (B) Structure of the BCS1 protein in the ATP‐bound form with one chain showing secondary structure with different parts differentially colour‐coded and the other chains of the heptamer shown as surfaces. Residues 1–28: possible mitochondrial translocation signal (structure unknown, added for illustrative purposes only); 29–49: transmembrane helix; 50–165: BCS1‐specific domain; 166–354: RecA‐like part of the ATPase domain and 355–418: helical bundle part of the ATPase domain. (C) Illustrates the frequency of the *BCS1L* phenotypes for each category of predicted secondary structure effect in relation to pathogenic variants identified in the patient cohort. Figures in brackets are patient study IDs. Scale of heatmap: frequency expressed as a ratio.

A total of 23 different pathogenic *BCS1L* variants were identified in the 33 individuals described in this study (Figure 1A, Table S4), including 9 novel disease‐causing variants: NM_001079866.1:c.‐50+358G>A, c.98G>A (p.Arg33Gln), c.487G>A (p.Glu163Lys), c.38A>G (p.Asn13Ser), c.688G>C (p.230Arg), c.785_786del CT (p.Ser262*), c.919C>T (p.Leu307Phe), c.1220_1220delC (p. Pro407Leufs*2) and c.1250T>C (p.Leu417Pro). Nineteen patients had homozygous pathogenic variants and the remaining 14 patients had compound heterozygous pathogenic variants (Table S4). All patients (*n* = 9) who were homozygous for the c.232A>G (p.Ser78Gly) variant presented at birth whilst those who were compound heterozygous for c.232A>G (p.Ser78Gly) with another variant (*n* = 4) presented within the first month of life. Those with other *BCS1L* pathogenic variants (*n* = 20), whether homozygous or compound heterozygous, presented from birth up to 7 years of age. Features consistent with GRACILE syndrome were more frequently reported in patients with homozygosity or compound heterozygosity for the c.232A>G (p.Ser78Gly) variant, whilst features such as movement disorders and seizures were predominantly observed in patients with pathogenic *BCS1L* variants other than c.232A>G (p.Ser78Gly) (Table 3). Allele frequencies of confirmed pathogenic variants of *BCS1L* using gnomAD v3.1.1 database accessed 13 June 2021, showed total pathogenic variant frequency = 0.001334057 ≈ 1:750 and total estimated lifetime risk = 1:561890. Variants listed in gnomAD as pathogenic or likely pathogenic were not included if no clinical confirmatory evidence of pathogenicity was available (Table S5).

**Table 3:** Phenotypic and survival data stratified into three groups, those with homozygous c.232A>G (p.Ser78Gly), compound heterozygous c.232A>G (p.Ser78Gly) or those with other pathogenic *BCS1L* gene variants.

*BCS1L* gene variants	Homozygous c.232A>G (p.Ser78Gly)	Compound heterozygous c.232A>G (p.Ser78Gly)	Other pathogenic *BCS1L* gene variants
Phenotype			
Age at disease onset	At birth	Birth‐29 days	Birth‐7 years (median: 3 days)
Lactic acidosis	9/9 (100%)	4/4 (100%)	13/17 (76%)
Hepatopathy	9/9 (100%)	4/4 (100%)	11/19 (58%)
Failure to thrive/feeding difficulties	8/8 (100%)	4/4 (100%)	15/20 (75%)
Proximal renal tubulopathy	7/7 (100%)	1/3 (33%)	16/20 (80%)
Growth restriction	9/9 (100%)	3/4 (75%)	7/17 (41%)
Sensorineural hearing loss	2/2 (100%)	2/2 (100%)	8/15 (53%)
Pili tori	0/1 (0%)	0/1 (0%)	4/13 (31%)
Leigh‐like phenotype	0/9 (0%)	1/4 (25%)	1/20 (5%)
Seizures	0/9 (0%)	1/4 (25%)	5/15 (25%)
Movement disorders	0/9 (0%)	0/4 (0%)	9/20 (25%)
Survival data			
Survival status ‐ alive	0/9 (0%)	2/3 (67%)	12/19 (63%)
Survival status ‐ deceased	9/9 (100%)	1/3 (33%)	7/19 (37%)
Early death	9/9 (100%)	1/3 (33%)	4/19 (21%)
Median time to death (range)	4 days (1 day–3 months)	1 month (single case)	2 years (1 month–14 years)

### Survival analysis

Fourteen of 33 patients were alive at the time of data analysis whilst 2 had been lost to follow‐up. Median survival time from disease onset to death for the whole cohort was 33 days (range 1 day to 14 years). The main cause of death was multi‐organ failure (*n* = 15/17, 88%) followed by sepsis (*n* = 2/17, 12%). The median survival time of patients with disease onset within the first month of life was 27 days (range 1 day–4.5 years), compared to 8 years (range 2–14 years) for those with disease onset after 1 month (Table 2).

**Figure 2:**
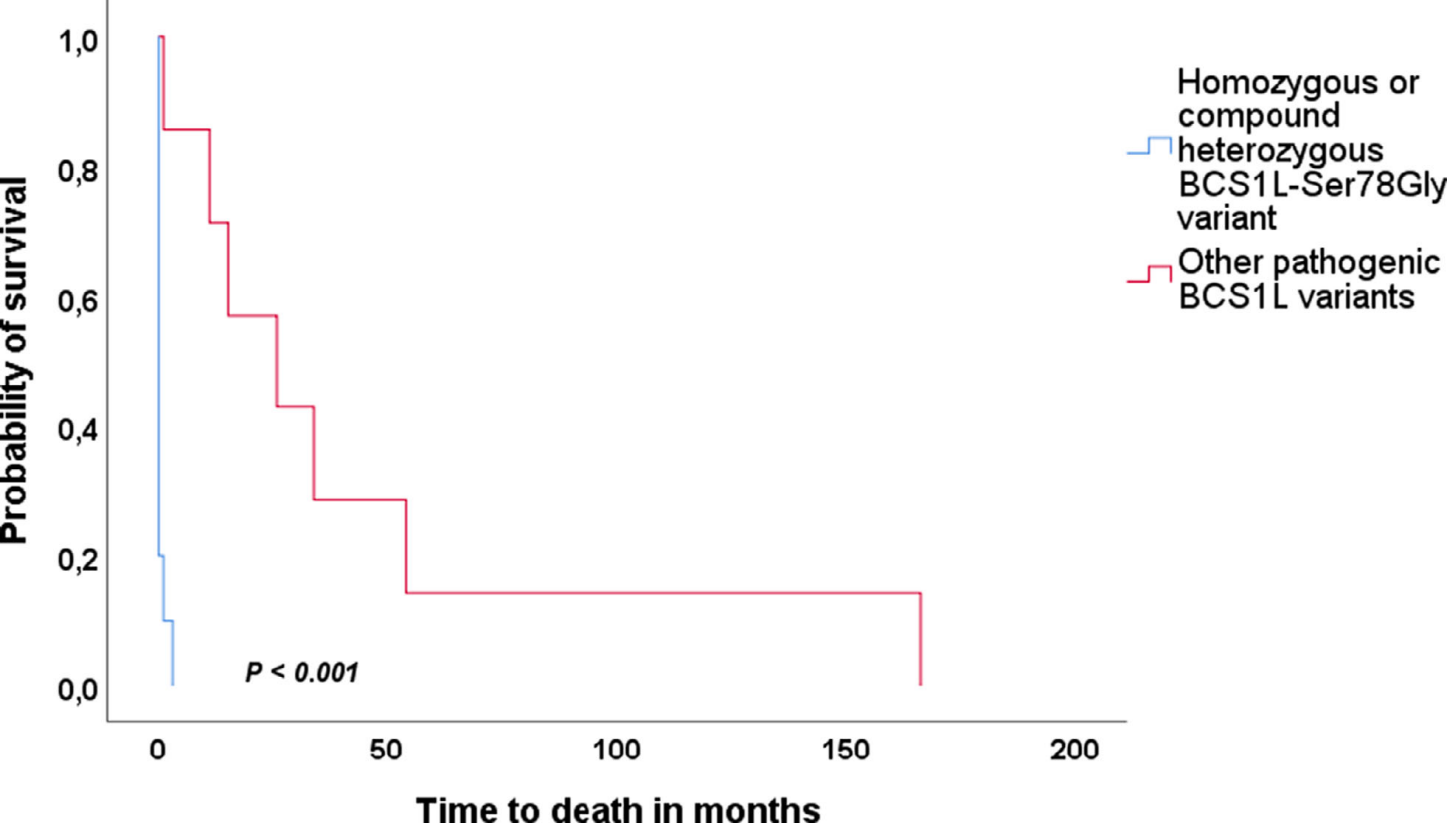
Kaplan–Meier curve comparing survival in patients with homozygous or compound heterozygous c.232A>G (p.Ser78Gly) and those with other pathogenic *BCS1L* variants. Patients with homozygous or compound heterozygous c.232A>G (p.Ser78Gly) variant had significantly (*p* < 0.001) worse survival as compared to those with other pathogenic *BCS1L* gene variants.

Survival analysis by genotype showed that the median survival time for patients homozygous for the c.232A>G (p.Ser78Gly) variant was 4 days (range 1 day–3 months), compared to 2 years (range 1 month–14 years) for those with other pathogenic *BCS1L* variants (Table 3). Further analysis revealed that patients homozygous or compound heterozygous for the c.232A>G (p.Ser78Gly) variant had significantly (*p* < 0.001) worse survival compared with those having other pathogenic *BCS1L* gene variants (Figure 2).

### Structural features

The consequences of the variants at the protein level were assessed in both ATP‐bound and unbound conformations (Figure 1B, interactive: https://michelanglo.sgc.ox.ac.uk/r/bcs1).

Three mutations were in regions that do not form a globular domain, namely p.Asn13Ser, p.Arg33Gln and p.Arg45Cys. Eight mutations occur in the domain that sits between the membrane and the ATP hydrolase domain, and four occur in the two parts of the hydrolase domain. The mutations were generally destabilising to both ATP‐bound and unbound forms, but with some differences and exceptions (Table S6).

Two variants (p.Gly230Arg and p.Cys252Tyr) are predicted to be extremely destabilising in both conformations (>+20 kcal/mol) due to severe clashes and most likely do not result in any folded protein, similar to the truncations. In support of this, these two mutations and the truncations are found solely as compound heterozygous variants in combination with structurally less severe mutations (Table S4, Figure 1C). It is anticipated that complete loss of function may be embryonically lethal as seen in knockout mice.^26^ A combination of one of these truncations and a splice variant that is predicted to affect protein expression is likely to have an effect due to insufficient protein abundance (patients 22, 23, 24, 32). The p.Leu417Pro variant may fall in the same category, but this cannot be predicted with confidence due to missing density in the apo form model. Other variants are predicted to be destabilising (p.Pro99Leu and p.Arg109Trp), affect one conformation more than the other (apo destabilising: p.Arg69Cys, p.Gly129Arg and p.Arg155Gln, ATP‐form destabilising: p.Leu307Phe), alter the balance of forces (p.Glu163Lys and p.Arg183His) and/or weaken oligomerisation (p.Ser78Gly). Counterintuitively, the combination of a loss of protein allele and a different allele does not result in the same phenotype as the patients homozygous for the latter alleles (patients 26 vs. 31; 17 & 33 vs. 14 &15), indicating it is not simply a case of the variants resulting in different concentrations of protein below the tolerance threshold accounting for the severity of symptoms.

p.Ser78Gly affects the interface (+2.6 kcal/mol) between different chains, but is not predicted to be destabilising, therefore may result in weaker oligomerisation.

Several of the affected residues are key to the conformational switch of the protein. Two mutations, p.Glu163Lys and p.Arg183His, are predicted to be neutral overall in both conformations, but substantially alter the chemical characteristics and are found in key positions for the conformational switch; specifically, the angle of the loop of Glu163 allows it to form a salt bridge with Arg184 in the apo form, but not in the ATP‐bound form.

## Discussion

We present the detailed description of 33 patients with confirmed pathogenic biallelic *BCS1L* variants, and demonstrate the breadth of clinical manifestations and the natural history of the disease. As far as we can ascertain, this is the largest cohort of patients with *BCS1L* disease so far described. Our studies also extend the phenotypic spectrum of the disease by identifying novel phenotypes other than the classic GRACILE and Björnstad syndromes.^12^, ^15^ Lastly, we show that it is possible to predict prognosis based on phenotypic and genetic factors, particularly age of onset and/or a genotype that includes the c.232A>G (p.Ser78Gly) variant.

We systematically reviewed our detailed clinical, laboratory, neuroimaging and genetic data, both at disease onset and later during the disease course. GRACILE and Björnstad syndromes are the most frequently reported phenotypes associated with pathogenic variants in *BCS1L*.^27^ Previously, 31 newborn infants have been diagnosed with GRACILE syndrome with the typical Finnish mutation and a very similar phenotype has been found to be caused by the Turkish mutation c.296C>T(p.Pro99Leu) and in a patient in New Zealand with a compound heterozygous mutation (Table S7). In the present study, only 13/33 (39%) fulfilled diagnostic criteria for classical GRACILE syndrome and 2/33 (6%) for classical Björnstad syndrome. The phenotypes of the other patients (55%, *n* = 18/33) fell into other categories as summarised in Table 1. Our study demonstrates the breadth of the phenotypic spectrum associated with *BCS1L‐*related disease and shows that it clearly includes features associated with mitochondrial dysfunction involving the central nervous system such as movement disorders, seizures and Leigh‐like phenotype. Amongst those with onset after the first month of life, we observed patients with a disease‐free period.

Interestingly, movement disorders including dystonia, athetosis and tremor, were frequently observed in our study cohort, regardless of the age of disease onset. Patients with mitochondrial disorders often show movement disorders associated with involvement of the basal ganglia.^28^ Although MRIs of the brain were available from only 10 patients, merely 12% of them showed changes in the basal ganglia. The patient with the most unusual movement disorder in this cohort (case 14) presented with paroxysmal exacerbation of ataxia with some subtle dystonic posturing that was precipitated by exercise and relieved by stress and not associated with basal ganglia lesions. In our series, 27% (*n* = 9) of the patients presented with movement disorders, in contrast to higher rates reported in other series of patients with mitochondrial disorders.^28^ Another consideration is that patients with *BCSL1‐*related disease have a high mortality in the neonatal period, before movement disorders can develop.

Since we were able to collect a large number of patients for such a rare disease, we could assess the prevalence of non‐classical clinical features and follow disease development. By stratifying patients into those with onset within the first month of life and those with later onset, we could identify clear phenotypic, genetic and prognostic differences. Features consistent with GRACILE syndrome such as lactic acidosis, hepatopathy, tubulopathy, growth failure and early death were more frequently reported in patients with disease onset within the first month of life as compared to those with later disease onset. In contrast, neurological features including movement disorders and seizures were more frequently reported in those with onset after the first month of life (Table 2).

Twenty‐three pathogenic *BCS1L* variants located throughout the gene were identified (Figure 1A). Genotype–phenotype correlation analysis was performed and revealed that the presence of the c.232A>G (p.Ser78Gly) variant (in homozygous or compound heterozygous state) was exclusively found in those with disease onset within the first month of life. However, other *BCS1L* variants were also reported in this age group. Further analysis showed that all those with homozygous c.232A>G (p.Ser78Gly) presented at birth with features consistent with GRACILE including early death. Those who were compound heterozygous for c.232A>G (p.Ser78Gly) and another variant presented within the first month of life with features consistent with GRACILE syndrome but with variable additional features including movement disorders and seizures. Patients with other pathogenic *BCS1L* variants could have a symptom‐free period, from birth to their first presentation, for as long as 7 years. Neurological features such as movement disorders and seizures were more frequently reported in patients with pathogenic variants in *BCS1L* other than c.232A>G (p.Ser78Gly), however features such as lactic acidosis, tubulopathy, hepatopathy and growth failure were also reported in this group. Further analysis revealed that the presence of c.232A>G (p.Ser78Gly), in homozygous or compound heterozygous state, was significantly (*p* < 0.001) associated with worse survival as compared to other pathogenic *BCS1L* variants. This clear genotype–phenotype correlation has not only an essential prognostic value, but also an important implication in genetic counselling, especially for families seeking prenatal diagnosis.

Respiratory chain analysis showed CIII deficiency (CII+CIII or CIII) in skeletal muscle (10/12), liver (*n* = 3/4) and fibroblasts (*n* = 1/3). Previous published studies reported CIII deficiency predominantly in the liver,^17^, ^27^ however, our study reveals that CIII deficiency was frequently identified also in muscle. These findings need to be interpreted with caution due to small sample size. Our study also showed that normal respiratory chain analysis does not exclude the diagnosis of *BCS1L* disease, as five of the patients in our cohort in whom respiratory chain analysis was performed (skeletal muscle *n* = 2, liver *n* = 1 and cultured skin fibroblasts *n* = 2) had normal results.

Our data confirmed that *BCS1L* disease comprises a continuum of clinical features rather than a set of separate clinical identities. Nevertheless, by grouping the patients into those with disease onset within the first month of life and those with later onset, we could identify clear phenotypic and prognostic differences. Our structural modelling data also provide evidence for some tentative genotypic–phenotypic correlations (Figure 1C). All patients with GRACILE syndrome were either homozygous for the p.Ser78Gly variant (cases 2–10 inclusive) or were compound heterozygous for the p.Arg56* truncating variant in combination with a frameshift or other deleterious variant (cases 24, 27, 32 and 33).

Our data also lead us to believe that *BCS1L* disease may be under‐diagnosed due to the presence of phenotypes such as movement disorders, seizures, isolated tubulopathy and later onset of the disease (after the neonatal period) that may not trigger the clinical suspicion of *BCS1L‐*related disease. Thus, the possibility of *BCS1L‐*related disease needs to be considered in phenotypes beyond the classical GRACILE and Björnstad syndromes, and this may improve early clinical recognition of the disease.

## Conflict of Interest

The authors declare no financial or other conflict of interest related to this work.

## Authors Contributions

O.H and S.R designed the study, were responsible for data collection, analysed the data and drafted the initial manuscript and approved the final manuscript as submitted. P.I, N.K, M.F, E.F, M.A, M.B, N.D, D.D, D.G, H.H, F.I, N.J, M.K, E.M, K.N, J.D.O.E, S.P, A.P, G.B., K.T, D.B, S.W, J.T, L.B and V.F were responsible for data acquisition and analysis, revising the manuscript critically and approving the final manuscript as submitted. All authors are responsible for accuracy and integrity of the work.

## Supporting information


**Table S1.** Major laboratory findings of patients included in this study cohort.

**Table S2.** Respiratory chain enzyme activities.

**Table S3.** Summary of neuroimaging findings.

**Table S4.** Genetic findings: *BCS1L* variants observed in the cohort.

**Table S5.** Allele frequencies of pathogenic variants of *BCS1L*.

**Table S6.** Predicted consequences of variants at the protein level assessed in both ATP‐bound and unbound conformations.

**Table S7.** Published cases with *BCS1L* mutations and clinical phenotypes (*n* = 87).
