# Supplementary material for: Expanding the phenotypic spectrum of BCS1L‐related mitochondrial disease
Source: Ann Clin Transl Neurol. 2021 Oct 18;8(11):2155–65. doi: 10.1002/acn3.51470 (PMC8607453; doi:10.1002/acn3.51470)
Supplement: Supplementary file 1 — Table S1. Major laboratory findings of patients included in this study cohort. [file ACN3-8-2155-s006.docx]

**Supplementary table 1**. Major laboratory findings of patients included in this study cohort

| **Laboratory findings** | **Number of patients with pathological findings** | |
| --- | --- | --- |
|  | **At presentation** | **Later** |
| ***1- Blood*** |  |  |
| Raised lactate | 16/24 (67%) | 26/30 (87%) |
| Hypoglycemia | 10/20 (50%) | 13/25 (52%) |
| Raised urea | 2/13 (15%) | 6/22 (27%) |
| Raised creatinine | 3/17 (18%) | 7/12 (58%)) |
| Low albumin | 4/15 (27%) | 10/17 (59%) |
| Raised aspartate aminotransferase | 15/20 (75%) | 15/21 (71%) |
| Raised alanine aminotransferase | 13/23 (56%) | 13/24 (54%) |
|  |  |  |
| ***2- Urine*** |  |  |
| Albuminuria | 9/11 (82%) | 9/18 (50%) |
| Glycosuria | 9/10 (90%) | 10/14 (71%) |
| Aminoaciduria | 16/17 (94%) | 18/20 (90%) |
| Organic aciduria | 14/18 (78%) | 18/20 (90%) |
|  |  |  |
| **3- *Cerebrospinal fluid*** |  |  |
| Raised lactate | 2/5 (40%) | NA |
| Raised protein | 1/4 (25%) | NA |
