# Supplementary material for: Expanding the phenotypic spectrum of BCS1L‐related mitochondrial disease
Source: Ann Clin Transl Neurol. 2021 Oct 18;8(11):2155–65. doi: 10.1002/acn3.51470 (PMC8607453; doi:10.1002/acn3.51470)
Supplement: Supplementary file 2 — Table S2. Respiratory chain enzyme activities. [file ACN3-8-2155-s004.docx]

**Supplementary table 2**. Respiratory chain enzyme activities

| **Case no.** | **Complex**  **I** | **Complex II** | **Complex II+III** | **Complex III** | **Complex IV** | **Comments** |
| --- | --- | --- | --- | --- | --- | --- |
| 1 | ND | ND | ND | ND | ND | Decreasd ATP production-muscle |
| 2 |  |  |  |  |  | ND |
| 3 |  |  |  |  |  | ND |
| 4 |  |  |  |  |  | ND |
| 5 |  |  |  |  |  | ND |
| 6 |  |  |  |  |  | ND |
| 7 |  |  |  |  |  | ND |
| 8 |  |  |  |  |  | ND |
| 9 |  |  |  |  |  | ND |
| 10 |  |  |  |  |  | ND |
| 11 | ↓ | ND | ↓ | ND | ↓ | Muscle |
| 12a | Normal | ND | Normal | ND | Normal | Liver |
| 12b | Normal | ND | Normal | ND | Normal | Muscle |
| 13 |  |  |  |  |  | ND |
| 14 | Normal | ND | ↓ | ND | Normal | Muscle |
| 15 | ND | ND | Normal | ND | Normal | Fibroblast |
| 16 |  |  |  |  |  | ND |
| 17 | ↓ | ND | ↓ | ND | Normal | Muscle |
| 18 | Normal | ND | Normal | ND | Normal | Muscle |
| 19 | ↓ | Normal | ND | ↓ | ND | Muscle |
| 20 |  |  |  |  |  | ND |
| 21 |  |  |  |  |  | ND |
| 22 | Normal | Normal | ND | ↓ | Normal | Muscle |
| 23 |  |  |  |  |  | ND |
| 24 | ND | ND | ND | ↓ | Normal | Muscle |
| 25 | ND | ND | ↓ | ↓ | Normal | Muscle |
| 26 |  |  |  |  |  | ND |
| 27a | ND | ND | ND | ↓ | ND | Muscle |
| 27b | ND | ND | ND | ↓ | ND | Liver |
| 28 |  |  |  |  |  | ND |
| 29 | ND | ND | ND | ↓ | ND | Liver |
| 30 |  |  |  |  |  | ND |
| 31 | ↓ | ↓ | ↓ | ↓ | Normal | Muscle |
| 32 | Normal | Normal | Normal | Normal | Normal | Fibroblast |
| 33a | ND | ND | ND | ↓ | ND | Liver |
| 33b | ND | ND | ND | ↓ | ND | Muscle |
| 33c | ND | ND | ND | ↓ | ND | Fibroblast |

ND: no data available, ↓: decreased
