# Supplementary material for: Expanding the phenotypic spectrum of BCS1L‐related mitochondrial disease
Source: Ann Clin Transl Neurol. 2021 Oct 18;8(11):2155–65. doi: 10.1002/acn3.51470 (PMC8607453; doi:10.1002/acn3.51470)
Supplement: Supplementary file 3 — Table S3. Summary of neuroimaging findings. [file ACN3-8-2155-s007.docx]

**Supplementary table 3**. Summary of neuro-imaging findings

| **Cerebral MRI findings** | **Number of patients (%)** |
| --- | --- |
| Abnormal cerebral MRI findings | 8/10 (80%) |
| T2 hyperintensities scattered in deep white matter | 6/8 (75%) |
| T2 hyperintensities in thalamus | 5/8 (62%) |
| T2 hyperintensities dentate nucleus | 2/8 (25%) |
| T2 hyperintensities globus pallidus and putamen | 1/8 (12%) |
| Severe delay in myelination | 1/8 (12%) |
| General brain atrophy | 1/8 (12%) |
| Bilateral thalamus atrophy | 5/8 (62%) |
