# Supplementary material for: Expanding the phenotypic spectrum of BCS1L‐related mitochondrial disease
Source: Ann Clin Transl Neurol. 2021 Oct 18;8(11):2155–65. doi: 10.1002/acn3.51470 (PMC8607453; doi:10.1002/acn3.51470)
Supplement: Supplementary file 4 — Table S4. Genetic findings: BCS1L variants observed in the cohort. [file ACN3-8-2155-s005.docx]

**Supplementary table 4**. Genetic findings: *BCS1L* variants observed in the cohort

| **Case no.** | **Allele 1** | **Allele 2** |
| --- | --- | --- |
| 1 | c.232A>G (p.Ser78Gly) | c.464G>A (p.Arg155Gln) |
| 2 | c.232A>G (p.Ser78Gly) | c.232A>G (p.Ser78Gly) |
| 3 | c.232A>G (p.Ser78Gly) | c.232A>G (p.Ser78Gly) |
| 4 | c.232A>G (p.Ser78Gly) | c.232A>G (p.Ser78Gly) |
| 5 | c.232A>G (p.Ser78Gly) | c.232A>G (p.Ser78Gly) |
| 6 | c.232A>G (p.Ser78Gly) | c.232A>G (p.Ser78Gly) |
| 7 | c.232A>G (p.Ser78Gly) | c.232A>G (p.Ser78Gly) |
| 8 | c.232A>G (p.Ser78Gly) | c.232A>G (p.Ser78Gly) |
| 9 | c.232A>G (p.Ser78Gly) | c.232A>G (p.Ser78Gly) |
| 10 | c.232A>G (p.Ser78Gly) | c.232A>G (p.Ser78Gly) |
| 11 | c.385G>A (p.Gly129Arg) | c.385G>A (p.Gly129Arg) |
| 12 | c.296C>T (p.Pro99Leu) | c.296C>T (p.Pro99Leu) |
| 13 | c.296C>T (p.Pro99Leu) | c.296C>T (p.Pro99Leu) |
| 14 | c.133 C>T (p.Arg45Cys) | c.133 C>T (p.Arg45Cys) |
| 15 | c.133 C>T (p.Arg45Cys) | c.133 C>T (p.Arg45Cys) |
| 16 | c.385G>A (p.Gly129Arg) | c.385G>A(p.Gly129Arg) |
| 17 | c.98G>A (p.Arg33Gln) | c.688G>C (p.Gly230Arg) |
| 18 | c.325C>T (p.Arg109Trp) | c.325C>T (p.Arg109Trp) |
| 19 | c.232A>G (p.Ser78Gly) | c.487G>A (p.Glu163Lys) |
| 20 | c.296C>T (p.Pro99Leu) | c.296C>T (p.Pro99Leu) |
| 21 | c.38A>G (p.Asn13Ser) | c.38A>G (p.Asn13Ser) |
| 22 | c.1220_1220delC (p.Pro407Leufs*2) | c.-50+358G>A |
| 23 | c.1220_1220delC (p.Pro407Leufs*2) | c.-50+358G>A |
| 24 | c.166C>T (p.Arg56*) | c.-50+388C>G, c.-50+405A>G,  reduction of mRNA expression |
| 25 | c.166C>T (p.Arg56*) | c.205C>T (p.Arg69Cys) |
| 26 | c.548G>A (p.Arg183His) | c.548G>A (p.Arg183His) |
| 27 | c.232A>G (p.Ser78Gly) | c.1250 T>C (p.Leu417Pro) |
| 28 | c.755G>A (p.Cys252Tyr) | c.919C>T (p.Leu307Phe) |
| 29 | c.232A>G (p.Ser78Gly) | c.413C>T (p.Thr138Met) |
| 30 | c.205C>T (p.Arg69Cys) | c.785_786del CT (p.Ser262*) |
| 31 | c.548G>A (p. Arg183His) | c.166C>T (p.Arg56*) |
| 32 | c.166C>T (p.Arg56*) | c.-50+388C>G, c.-50+405A>G,  reduction of mRNA expression |
| 33 | c.133 C>T (p.Arg45Cys) | c.166C>T (p.Arg56*) |

Novel variants identified in this study are highlighted in red. Ref Seq used: NM_001079866.2
