# Supplementary material for: Expanding the phenotypic spectrum of BCS1L‐related mitochondrial disease
Source: Ann Clin Transl Neurol. 2021 Oct 18;8(11):2155–65. doi: 10.1002/acn3.51470 (PMC8607453; doi:10.1002/acn3.51470)
Supplement: Supplementary file 5 — Table S5. Allele frequencies of pathogenic variants of BCS1L. [file ACN3-8-2155-s003.docx]

**Supplementary table 5**. Allele frequencies of pathogenic variants of *BCS1L.* Allele frequencies are listed for confirmed pathogenic variants listed in the Gnomad v3.1.1 database accessed 13 June 2021.Total pathogenic variant frequency = 0.001334057 ≈ 1:750. Total estimated lifetime risk = 1:561890. Variants listed in Gnomad listed as pathogenic or likely pathogenic were not included if no clinical confirmatory evidence of pathogenicity was available.

| **Variant** | **Allele frequency** | **Reference** |
| --- | --- | --- |
| c.98G>A; p.Arg33Gln | 2.62864E-05 | This paper |
| c.166C>T;p.Arg56Ter | 0.000282545 | Visapää 2002^(1)^ |
| c.205C>T; p.Arg69Cys | 0.000105154 | This paper |
| c.217C>T;p.Arg73Cys | 5.25631E-05 | Fernandez-Vizarra 2007^(2)^ |
| c.232A>G;p.Ser78Gly | 0.000394317 | Fellman 1998^(3)^ |
| c.325C>T;p.Arg109Trp | 7.23246E-05 | Olahova 2019^(4)^ |
| c.341G>T;p.Arg114Leu | 1.97135E-05 | Hinson 2007^(5)^ |
| c.399delA;p.Glu133Aspfs*25 | 7.2279E-05 | Tegelberg 2017^(6)^ |
| c.431G>A;p.Arg144Gln | 1.31392E-05 | Visapää 2002^(1)^ |
| c.487G>A;p.Glu163Lys | 1.31401E-05 | This paper |
| c.547C>T; p.Arg183Cys | 7.22819E-05 | Fernandez-Vizarra 2007^(2)^ |
| c.548G>A; p.Arg183His | 6.57151E-06 | Hinson 2007^(5)^ |
| c.550C>T;p.Arg184Cys | 0.000111707 | Fernandez-Vizarra 2007/ Hinson 2007^(2, 5)^ |
| c.556C>T;p.Arg186* | 6.56918E-06 | Zhang 2015^(7)^ |
| c.871C>T;p.Arg291Ter | 4.60296E-05 | Hinson 2007^(5)^ |
| c.904C>G;p.Gln302Glu | 1.3146E-05 | Hinson 2007^(5)^ |
| c.917G>A;p.Arg306His | 1.31503E-05 | Zhang 2015^(7)^ |
| c.1057G>A;p.Val353Met | 1.31404E-05 | de Lonlay 2001^(8)^ |
