# Supplementary material for: Expanding the phenotypic spectrum of BCS1L‐related mitochondrial disease
Source: Ann Clin Transl Neurol. 2021 Oct 18;8(11):2155–65. doi: 10.1002/acn3.51470 (PMC8607453; doi:10.1002/acn3.51470)
Supplement: Supplementary file 6 — Table S6. Predicted consequences of variants at the protein level assessed in both ATP‐bound and unbound conformations. [file ACN3-8-2155-s001.docx]

**Supplementary table 6:** Predicted consequences of variants at the protein-level assessed in both ATP-bound and unbound conformations

| Mutation | Location | apo ∆∆G  [kcal/mol] | bound ∆∆G  [kcal/mol] | apo ∆∆G (interface)  [kcal/mol] | bound ∆∆G (interface)  [kcal/mol] | Predicted effect |
| --- | --- | --- | --- | --- | --- | --- |
| p.Asn13Ser | N-terminal end | nan | nan | nan | nan | **Potentially disrupts mitochondrial translocation signal.** Residues 2-28 do not match the signature of a canonical mitochondrial translocation signal, but this is absent in the density, which may suggest it is disordered or was cleaved off. |
| p.Arg33Gln | Transmembrane | 0.2 | 0.3 | 0.0 | 0.0 | **Potentially disrupts membrane anchoring.**  Arginines, are positively charged residues, which when situated at the start of a membrane spanning region may bind a phosphate head of the lipid bilayer. Arg33 and Lys34, next to it, are outwards facing positively charged residues in the transmembrane helix close to the intermembrane space. However, this is at odds with the 30° inwards tilt and membrane thickness, indicating that these two residues must be binding something and/or distorting the membrane. |
| p.Arg45Cys | Transmembrane | 2.3 | 2.0 | 1.4 | -1.0 | **Potentially disrupts membrane anchoring.**  Like Arg33 this is a charged residue within the transmembrane helix, but near the edge with the matrix. It is flanked by Arg45 and both face outwards, a helix turn further on, are methionine and tyrosine, the last two hydrophobic residues in the helix. This cannot be a diarginine motif for ER retention as mitochondrial protein are translocated from the cytoplasm, not via the ER |

| p.Arg56* | BCS1 specific domain | nan | nan | nan | nan | **Truncation.**  This truncation is unstructured |
| --- | --- | --- | --- | --- | --- | --- |

| p.Arg69Cys | BCS1 specific domain | 6.4 | 1.9 | 0.1 | 0.0 | **Apo destabilised.**  Destabilising due to loss of surface salt bridges with Glu96 on a nearby sheet —Apo structure only |
| --- | --- | --- | --- | --- | --- | --- |
| p.Ser78Gly | BCS1 specific domain | 1.8 | 2.6 | 2.2 | 0.5 | **Interface disrupted.**  Ser78 hydrogen bonds with Arg117 forming the core of the domain, this loss of bonding (+6 kcal/mol) is compensated by better backbone torsions (-5 kcal/mol), but weakens the interface with a nearby chain (+2 kcal/mol) |
| p.Pro99Leu | BCS1 specific domain | 7.4 | 9.9 | 0.1 | 0.0 | **Strongly destabilised.**  Pro99 is at the end of a sheet and before a turn, the mutation is deleterious (+7 kcal/mol), but due to clashes and not torsion |
| p.Arg109Trp | BCS1 specific domain | 5.7 | 7.5 | 0.0 | 0.0 | **Strongly destabilised.**  Arg109 forms a surface salt-bridge to Glu153. The change to a bulky hydrophobic residue is predicted to be destabilising (+6 kcal/mol) due to electrostatic and steric clashes |
| p.Gly129Arg | BCS1 specific domain | 9.7 | 1.9 | 3.3 | 0.3 | **Apo destabilised.**  Hydrogen donor residue of a &beta;-turn with Leu126. Highly destabilising (+10 kcal/mol) due to several steric clashes (Pauli repulsion forces) |
| p.Thr138Met | BCS1 specific domain | 3.1 | -1.6 | 2.0 | 2.3 | **Mildly destabilised.**  The mutation on this sheet residue is predicted to be destabilising (+3 kcal/mol) due to some steric clashes, which also affect interchain binding |
| p.Arg155Gln | BCS1 specific domain | 4.9 | 3.9 | -0.6 | -1.7 | **Apo destabilised.**  Arg155 founds a salt-bridge with the next residue along in the helix, Glu156, and the lower half of the sidechain interacts with another chain. This is predicted to be destabilising (+5 kcal/mol) due to steric clashes and worse hydrogen bonding |
| p.Glu163Lys | BCS1 specific domain | -0.2 | 0.1 | 0.1 | 0.0 | **Dynamic switch hindered.**  Glu163 forms a salt bridge with Arg184. Overall it is predicted to be energetically neutral but with a large trade off of worse electrostatic clashes with improved backbone torsions (similarly to Arg183His) |
| p.Arg183His | RecA-like domain | -1.9 | 5.9 | 1.3 | 1.8 | **Dynamic switch hindered.**  This residue is part of a four arginine repeat (unusual), which form various salt bridges, but are out of binding distance to the magnesium. This is predicted to be energetically neutral, but there is a large trade off of worse electrostatic clashes with improved backbone torsions (similarly to Glu163Lys) |
| p.Gly230Arg | RecA-like domain | 24.7 | 47.1 | 0.3 | 0.5 | **Unfolded.**  This residue disrupts the active site (over +20 kcal/mol), likely abolishing the ATP hydrolysis |
| p.Cys252Tyr | RecA-like domain | 28.4 | 12.8 | 0.2 | 0.7 | **Unfolded.**  The mutation of a small residue in the core of the RacA-like domain to a larger one is highly destabiling (over 20 kcal/mol) |

| p.Ser262* | RecA-like domain | nan | nan | nan | nan | **Truncation.**  This truncation is unlikely to be unstructured |
| --- | --- | --- | --- | --- | --- | --- |

| p.Leu307Phe | RecA-like domain | nan | 11.4 | nan | 0.6 | **ATP-bound destabilised.**  This residue in a mobile loop that is likely close to the active site (modelled loop: 14 Å away and buried). This region shift significantly during translocation. |
| --- | --- | --- | --- | --- | --- | --- |
| p.Leu417Pro | helical bundle domain | nan | 23.6 | nan | 0.0 | **Unfolded.**  The last three residues are LeuArgArg and are structural in the ATP-bound form, but only Leu is present in the Apo structure. The leucine sidechain is structural in both and the terminal Arg419 forms a salt-bridge in ATP-bound. It is likely to be as destabilising in the Apo form that in the ATP form. |
