# Supplementary material for: Expanding the phenotypic spectrum of BCS1L‐related mitochondrial disease
Source: Ann Clin Transl Neurol. 2021 Oct 18;8(11):2155–65. doi: 10.1002/acn3.51470 (PMC8607453; doi:10.1002/acn3.51470)
Supplement: Supplementary file 7 — Table S7. Published cases with BCS1L mutations and clinical phenotypes (n = 87). [file ACN3-8-2155-s002.docx]

**Supplementary table 7.** Published cases with *BCS1L* mutations and clinical phenotypes

(n=87)

| **Mutation** | **αα* change** | **Number of cases** | **Phenotype** | **Country of origin** | **Reference** |
| --- | --- | --- | --- | --- | --- |
| **Homozygous mutations** | | | | | |
| c.232A>G | Ser78Gly | 31 | GRACILE syndrome (died at age median; IQR* 31; 2-63 d) | Finland, Sweden (Finnish ancestors) | Fellman 1998^(3)^, 2008^(9)^, 2012^(10)^, Visapää 2002^(1)^ Kotarsky 2010^(11)^ |
| c.296C>T | Pro99Leu | 4 | GRACILE-like disease, iron accumulation not assessed (died 6mth, 2y, ?, 37 d) | Turkey | De Lonlay 2001^(8)^, Kasapkara 2014^(12)^, Serdarglu 2016^(13)^ |
| c.325C>T | Arg109Trp | 1 | GRACILE-like disease, hepatopathy, hyperinsulinism, iron accumulation not assessed (died 4 mths) | Bangladesh | Olahova 2019^(4)^ |
| c.830G>A | Ser277Asn | 2 | Neonatal tubulopathy, hepatic failure, encephalopathy (died 3 mth, >9y) | Turkey | de Lonlay 2001^(8)^ |
| c.148 A>G | Thr50Ala | 1 | Neonatal hepatopathy, infantile psychomotor retardation, failure to thrive, hypotonia, lactic acidosis, tubulopathy, mild sensorineural hearing loss (alive >4y) | Spain | Blazquez 2008^(14)^ |
| c.548G>A | Arg183His | 8 | Björnstad syndrome | Norway | Hinson 2007^(5)^ |
| c.917G>A | Arg306His | 1 | Björnstad syndrome | Norway | Hinson 2007^(5)^ |
| c.901T>A | Tyr301Asn | 5 | Björnstad syndrome | Pakistan | Siddiqi 2013^(15)^ |
| c.385G>A | Gly129Arg | 1  9 | Muscle weakness and optic atrophy (adult onset, but floppy newborn)  Neonatal lactic acidosis, later neurocognitive deficits (pediatric) | Kenya  Saudi Arabia | Tuppen 2010^(16)^  Al-Owain 2013^(17)^ |
| c.142A>G | Met48Val | 3 | Neonatal lactacidosis, tubulopathy, rachitis, hearing impairment, neurocognitive deficits, liver dysfunction (pediatric) | Turkey | Jackson 2016^(18)^ |
| c.296C>T | Pro99Leu | 1 | Intrauterine growth restriction, metabolic acidosis, neonatal hepatopathy, hearing loss. Died 4mth | Turkey | Akduman 2016^(19)^ |
| **Compound heterozygous mutations** | | | | | |
| c.232A>G  c.431G>A | Ser78Gly  Arg144Gln | 1 | Neonatal lactacidosis, tubulopathy, liver dysfunction (died 15 wks) | UK | Visapää 2002^(1)^ |
| c.166C>T  c.980T>C | Arg56*  Val327Ala | 1 | Neonatal lactacidosis, seizures, tubulopathy (died 2d) | UK | Visapää 2002^(1)^ |
| -c.588T>A  c.321G>T | Truncated protein | 1 | Neonatal lactacidosis, tubulopathy, hepatopathy (died 6 wk) | UK | Visapää 2002^(1)^ |
| c.464G>C c.1057G>A | Arg155Pro  Val353Met | 1 | Lactic acidosis, liver dysfunction, renal tubulopathy, neurological symptoms (lived> 0.5y) | Turkey | de Lonlay 2001^(8)^ |
| c.133C>T c.166C>T | Arg45Cys  Arg56* | 3 | Lactic acidosis, liver dysfunction, renal tubulopathy, iron overload, neurological symptoms (died 3wk, 3 mths, 6 mths) | Spain | De Meirleir 2003^(20)^  Ramos-Arroyo 2009^(21)^ |
| c.550C>T  c.103G>C | Arg184Cys  Gly35Arg | 1 | Mild Björnstad syndrome | Norway | Hinson 2007^(5)^ |
| ?  c.917G>A | IVS2+1g>t  Arg306His | 1 | Björnstad syndrome | Norway | Hinson 2007^(5)^ |
| c.917G>A  c.341G>T | Arg306His  Arg114Trp | 1 | Björnstad syndrome | Norway | Hinson 2007^(5)^ |
| c.871C>T  c C>G | Arg291*  Gln302Glu | 1 | Björnstad syndrome | Norway | Hinson 2007^(5)^ |
| c.217C >T  c.1102T>A | Arg73Cys  Phe368Ile | 1 | Infantile progressive encephalopathy | Italy | Fernandez-Vizarra 2007^(2)^ |
| c.547C>T c.550C>T | Arg183Cys  Arg184Cys | 1 | Infantile progressive encephalopathy | Marocco | Fernandez-Vizarra 2007^(2)^ |
| c.166C>T  g.1181A>G  g.1164C>G | Arg56*  Splicing variant | 1 | Neonatal hepatopathy, later tubulopathy, lactic acidosis (died 11mth) | Spain | Gil-Borlado 2009^(22)^ |
| -c.588T>A  c.166C>T | Splice  Arg56* | 1 | GRACILE-like, hyperinsulinism  (iron accumulation ND, died 3 mth) | New Zealand | Lynn 2012^(23)^ |
| c.556C>T  c.916C>T | Arg186*  Arg306Cys | 2 | Hearing loss, brittle hair (Björnstad) | China | Zhang 2015^(7)^ |
| c.399delA  c.306A > T | Glu133Aspfs*25  Cryptic splice site | 1 | Neonatal lactacidosis, hypotonia, encephalopathy,failure to thrive (died 13 mth) | Sweden | Tegelberg 2017^(6)^ |
| c.548G>A  c.1061_1062  insCTA | Arg183His  Gly354delins  GlyTyr | 1 | Mild lactic acidosis (at 7 mths), psychomotor retardation, infantile spasms, liver dysfunction | China | Liu 2020^(24)^ |
| c.550C>T  c.838C>T | Arg184Cys  p.Leu280Phe | 1 | Failure to thrive, bilateral sensorineural hearing loss, hypotonia, global developmental delay, encephalopathy, nystagmus, brittle hair, died 29mth | USA | Baker et al 2019^(25)^ |

**References**

1. Visapää I, Fellman V, Vesa J, Dasvarma A, Hutton JL, Kumar V, et al. GRACILE syndrome, a lethal metabolic disorder with iron overload, is caused by a point mutation in BCS1L. American journal of human genetics. 2002;71(4):863-76.

2. Fernandez-Vizarra E, Bugiani M, Goffrini P, Carrara F, Farina L, Procopio E, et al. Impaired complex III assembly associated with BCS1L gene mutations in isolated mitochondrial encephalopathy. Human molecular genetics. 2007;16(10):1241-52.

3. Fellman V, Rapola J, Pihko H, Varilo T, Raivio KO. Iron-overload disease in infants involving fetal growth retardation, lactic acidosis, liver haemosiderosis, and aminoaciduria. Lancet (London, England). 1998;351(9101):490-3.

4. Oláhová M, Ceccatelli Berti C, Collier JJ, Alston CL, Jameson E, Jones SA, et al. Molecular genetic investigations identify new clinical phenotypes associated with BCS1L-related mitochondrial disease. Human molecular genetics. 2019;28(22):3766-76.

5. Hinson JT, Fantin VR, Schönberger J, Breivik N, Siem G, McDonough B, et al. Missense mutations in the BCS1L gene as a cause of the Björnstad syndrome. The New England journal of medicine. 2007;356(8):809-19.

6. Tegelberg S, Tomašić N, Kallijärvi J, Purhonen J, Elmér E, Lindberg E, et al. Respiratory chain complex III deficiency due to mutated BCS1L: a novel phenotype with encephalomyopathy, partially phenocopied in a Bcs1l mutant mouse model. Orphanet journal of rare diseases. 2017;12(1):73.

7. Zhang J, Duo L, Lin Z, Wang H, Yin J, Cao X, et al. Exome sequencing reveals novel BCS1L mutations in siblings with hearing loss and hypotrichosis. Gene. 2015;566(1):84-8.

8. de Lonlay P, Valnot I, Barrientos A, Gorbatyuk M, Tzagoloff A, Taanman JW, et al. A mutant mitochondrial respiratory chain assembly protein causes complex III deficiency in patients with tubulopathy, encephalopathy and liver failure. Nature genetics. 2001;29(1):57-60.

9. Fellman V, Lemmelä S, Sajantila A, Pihko H, Järvelä I. Screening of BCS1L mutations in severe neonatal disorders suspicious for mitochondrial cause. Journal of human genetics. 2008;53(6):554-8.

10. Fellman V. [GRACILE syndrome--a severe neonatal mitochondrial disorder]. Duodecim; laaketieteellinen aikakauskirja. 2012;128(15):1560-7.

11. Kotarsky H, Karikoski R, Mörgelin M, Marjavaara S, Bergman P, Zhang DL, et al. Characterization of complex III deficiency and liver dysfunction in GRACILE syndrome caused by a BCS1L mutation. Mitochondrion. 2010;10(5):497-509.

12. Kasapkara Ç S, Tümer L, Ezgü FS, Küçükçongar A, Hasanoğlu A. BCS1L gene mutation causing GRACILE syndrome: case report. Renal failure. 2014;36(6):953-4.

13. Serdaroğlu E, Takcı Ş, Kotarsky H, Çil O, Utine E, Yiğit Ş, et al. A Turkish BCS1L mutation causes GRACILE-like disorder. The Turkish journal of pediatrics. 2016;58(6):658-61.

14. Blázquez A, Gil-Borlado MC, Morán M, Verdú A, Cazorla-Calleja MR, Martín MA, et al. Infantile mitochondrial encephalomyopathy with unusual phenotype caused by a novel BCS1L mutation in an isolated complex III-deficient patient. Neuromuscular disorders : NMD. 2009;19(2):143-6.

15. Siddiqi S, Siddiq S, Mansoor A, Oostrik J, Ahmad N, Kazmi SA, et al. Novel mutation in AAA domain of BCS1L causing Bjornstad syndrome. Journal of human genetics. 2013;58(12):819-21.

16. Tuppen HA, Fehmi J, Czermin B, Goffrini P, Meloni F, Ferrero I, et al. Long-term survival of neonatal mitochondrial complex III deficiency associated with a novel BCS1L gene mutation. Molecular genetics and metabolism. 2010;100(4):345-8.

17. Al-Owain M, Colak D, Albakheet A, Al-Younes B, Al-Humaidi Z, Al-Sayed M, et al. Clinical and biochemical features associated with BCS1L mutation. Journal of inherited metabolic disease. 2013;36(5):813-20.

18. Jackson CB, Bauer MF, Schaller A, Kotzaeridou U, Ferrarini A, Hahn D, et al. A novel mutation in BCS1L associated with deafness, tubulopathy, growth retardation and microcephaly. European journal of pediatrics. 2016;175(4):517-25.

19. Akduman H, Eminoglu T, Okulu E, Erdeve O, Atasay B, Arsan S. A neonate presenting with GRACILE syndrome and Bjornstad phenotype associated with BCS1L mutation. Genetic counseling (Geneva, Switzerland). 2016;27(4):509-12.

20. De Meirleir L, Seneca S, Damis E, Sepulchre B, Hoorens A, Gerlo E, et al. Clinical and diagnostic characteristics of complex III deficiency due to mutations in the BCS1L gene. American journal of medical genetics Part A. 2003;121a(2):126-31.

21. Ramos-Arroyo MA, Hualde J, Ayechu A, De Meirleir L, Seneca S, Nadal N, et al. Clinical and biochemical spectrum of mitochondrial complex III deficiency caused by mutations in the BCS1L gene. Clinical genetics. 2009;75(6):585-7.

22. Gil-Borlado MC, González-Hoyuela M, Blázquez A, García-Silva MT, Gabaldón T, Manzanares J, et al. Pathogenic mutations in the 5' untranslated region of BCS1L mRNA in mitochondrial complex III deficiency. Mitochondrion. 2009;9(5):299-305.

23. Lynn AM, King RI, Mackay RJ, Florkowski CM, Wilson CJ. BCS1L gene mutation presenting with GRACILE-like syndrome and complex III deficiency. Annals of clinical biochemistry. 2012;49(Pt 2):201-3.

24. Liu X, Zhang Y, Liang J, Yang S, Chen C. A novel mutation in the ubiquinol-cytochrome c reductase synthesis-like gene associated with complex III deficiency and Björnstad syndrome: A case report. Medicine. 2020;99(44):e23026.

25. Baker RA, Priestley JRC, Wilstermann AM, Reese KJ, Mark PR. Clinical spectrum of BCS1L Mitopathies and their underlying structural relationships. American journal of medical genetics Part A. 2019;179(3):373-80.
